# Polymorphism of a Highly Asymmetrical Triacylglycerol in Milk Fat: 1-Butyryl 2-Stearoyl 3-Palmitoyl-glycerol

**DOI:** 10.1021/acs.cgd.2c00713

**Published:** 2022-09-13

**Authors:** Yoga Pratama, Sam Burholt, Daniel L. Baker, Amin Sadeghpour, Elena Simone, Michael Rappolt

**Affiliations:** † School of Food Science and Nutrition, Food Colloids and Bioprocessing Group, University of Leeds, Leeds LS2 9JT, United Kingdom; ‡ Department of Food Technology, Faculty of Animal and Agricultural Sciences, Diponegoro University, Semarang 50275, Indonesia; § Diamond-Leeds Small Angle X-ray Scattering Facility, Didcot Oxfordshire OX11 0DE, United Kingdom; ∥ School of Physics and Astronomy, University of Leeds, Leeds LS2 9JT, United Kingdom; ⊥ Department of Applied Science and Technology, Politecnico di Torino, Torino 10129, Italy

## Abstract

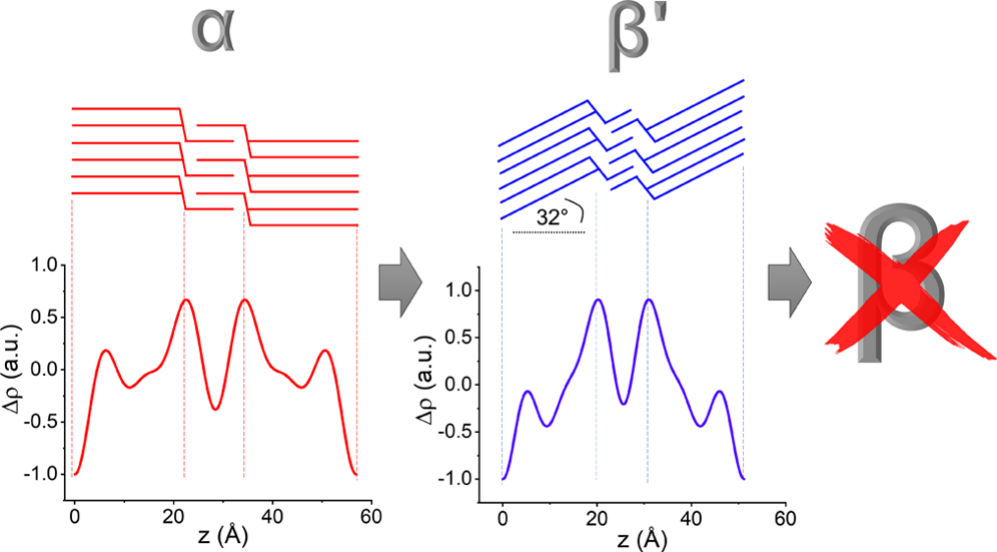

Milk fat has more than 200 triacylglycerols (TAGs), which play a pivotal role in its crystallization behavior. Asymmetrical TAGs containing short butyryl chains contribute to a significant portion of milk fat TAGs. This work aims to elucidate the crystallization behavior of asymmetrical milk fat TAGs by employing the pure compound of 1-butyryl 2-stearoyl 3-palmitoyl-glycerol (BuSP). The structural evolution of BuSP after being cooled down to 20 °C from the melt is evaluated by small- and wide-angle X-ray scattering (SAXS and WAXS) and differential scanning calorimetry (DSC). The temporal structural observation shows that BuSP crystallizes into the α-form with short and long spacings of 4.10 and 56.9 Å, respectively, during the first hour of isothermal hold at 20 °C. The polymorphic transformation of the α to β′ phase occurred after 4 h of isothermal hold, and the β′- to α-form fraction ratio was about 70:30 at the end of the isothermal experiment (18 h). Pure β′-form X-ray patterns are obtained from the BuSP powder with short spacings of 4.33, 4.14, and 3.80 Å, while the long spacing of 51.2 Å depicts a three-chain-length lamellar structure with a tilt angle of 32°. Corresponding DSC measurements display that BuSP crystallizes from the melt at 29.1 °C, whereas the melting of α- and β′-forms was recorded at 30.3 and 47.8 °C, respectively. In the absence of the β-form, the β′-polymorph is the most stable observed form in BuSP. This work exemplarily explains the crystallization behavior of asymmetrical milk fat TAGs and thus provides new insights into their role in overall milk fat crystallization.

## Abstract 2

The crystallization behavior of a pure asymmetrical TAG of milk fat is discussed in great detail, where the temporal observation shows a slow polymorphic transformation, with β′ as the most stable form.

## Introduction

Milk fat is one of the most complex fats found in nature. More than 200 different triacylglycerol (TAG) species have been reported^1^ to make up 98% of the total composition of milk fat.^2^ Additionally, milk fat contains some minor components such as sterols, phospholipids, free fatty acids, and mono- and di-acylglycerols.^3^ The wide array of TAGs in milk fat contains 13 major fatty acids, whose contribution to the total composition is each higher than 1% (w/w). Nine fatty acids are saturated (C_4:0_, C_6:0_, C_8:0_, C_10:0_, C_12:0_, C_14:0_, C_15:0_, C_16:0_, and C_18:0_), whereas four are unsaturated (C_14:1_, C_16:1_, C_18:1_, and C_18:2_).^1^ In addition to its large number of TAGs, the milk fat TAG composition is known to be influenced by a number of factors such as breed, stage of lactations, seasons, and geographical location where milking species are grown as well as the type of feed.^4−10^ Nevertheless, the compositional differences in milk fat among different milking species, for instance, cow *vs* buffalo *vs* goat, are greater than that of interspecies variation.^11−13^ As an example, in our previous study, we found that 20 out of 37 identified TAGs differ significantly in their proportion between cow and buffalo milk fat.^14^

Because of their complex nature, milk fats exhibit a broad melting range and complex crystallization behavior, with multiple crystal structures forming.^15−19^ In fact, at least five different molecular chain packing arrangements are known, corresponding to α, γ (sub-α), β′-1, β′-2, and β packing configurations. On the other hand, numerous stacking architectures have been observed, which fall into two main lamellar stacking types. First, double-chain-length (2L) structures with the *d*-spacing ranging from 39 to 48 Å and triple-chain-length (3L) structures with *d*-spacings between 53 and 73 Å.^14,16^ Moreover, the polymorphic behavior during milk fat crystallization is strongly influenced by processing factors such as cooling rate, isothermal holding time, and the presence of shear.^15,20−23^

The structure of milk fat crystals is essential as it directly affects the physical properties of fat-based dairy products, such as their melting characteristics, mouthfeel, stability, and spreadability.^24^ The chain packing information that denote the polymorph types are commonly obtained from wide-angle X-ray scattering (WAXS) measurements. In addition, measurements in the small-angle X-ray scattering (SAXS) regime provide lamellar thickness of 2L and 3L stacking repeat, which in turn allows one to approximately identify which types of TAGs contribute to a given polymorph structure. Thus, an accurate identification of the chain packing with its accompanying stacking type (2L or 3L) allows pinpointing the TAGs that are prevalent in a specific crystal structure. Finally, the identification of TAG self-assembled structures is important to understand how milk fat crystallizes and offers great opportunities for controlling and further tailoring milk fat processing, for example, by manipulating the proportions of TAGs to obtain the polymorph and kinetics of nucleation and growth that suits a specific dairy product best.

In single polymorphic TAG samples, it is straightforward to associate the lamellar stackings to the given chain packing type. However, in mixtures of polymorphic forms, the association of different lamellar thicknesses with their corresponding chain packing can be tricky. As an illustration, a straightforward association of the 2L (46 Å) and a 3L (72 Å) lamellar structure with a hexagonal chain packing could be unanimously accepted because they were observed in a pure α-system when the milk fat was cooled at a −3 °C/min rate.^21^ Our previous study also observed the same finding with 48.4 and 72.8 Å stacking distances, corresponding to the α-polymorph after cooling at a −2 °C/min rate.^14^ Similarly, a very unstable γ-form was the only observed structure upon the rapid quenching of milk fat to −8 °C. Thus, also here, the two stacking configurations with 70 and 47 Å lattice spacings (3L and 2L) could be readily attributed to the same packing type, the γ-phase.^25^

On the other hand, association of the lamellar structures with β′- and β-polymorphic packing in milk fat is not as straightforward. These polymorphs are rarely observed in their pure form in milk fat, but they often coexist with each other or with the metastable α-phase. For example, Lopez et al.^21^ attributed the lattice structure with *d*-spacings of 40–41.5 Å to the β′-polymorph with a 2L architecture. While a crystal structure with a *d*-spacing of 67 Å could be attributed to a 3L stacking structure, it could only be tentatively assigned to the β′-form due to the coexistence with an α-polymorph. In our studies, we recently confirmed that the long spacing of 67 Å corresponds to the β′-phase after conducting a long isothermal experiment, diminishing most of the α-phase and, additionally, being able to determine the electron density profile of the β′-form.^14^ The correct phase identification is even more difficult concerning the β-polymorph. Indeed, its existence in milk fat is still a subject of ongoing debate. Some studies did not observe the β-form at all,^15,20^ while other studies reported the presence of this polymorph as trace and or in coexistence with other crystal structures.^14,21,23,26^ Indeed, the full understanding of this triclinic system and its corresponding lamellar configurations in milk fat is still lacking.

Having summarized most of the key lamellar thicknesses of milk fat crystals above, one repeat distance of particular interest has a *d*-spacing of 53 Å. The nature of this particular structure of milk fat is still fairly unclear, unlike those of the 3L structures of 67–73 Å that are associated with long-chain milk TAGs, containing unsaturated fatty acid(s) or the 2L structures of 39–48 Å that are associated with long-chain fully saturated TAGs.^14,16^ We observed this 53 Å lamellar structure in our previous study^14^ and speculated that it may correspond to a β-crystal polymorph. In fact, a similar lattice spacing (54 Å) was also reported by Lopez et al.,^21^ despite no polymorph-type association being made. However, both studies agree that at 53–54 Å length, the stacking type should be that of a triple-layered chain structure. This unknown polymorph has a very short *d*-spacing for a 3L structure, especially when compared to the well-known α-3L (72 Å) and β′-3L (67 Å). Therefore, we concluded that this stacking configuration can only be a result of asymmetrical TAGs containing the very short butyric (C_4:0_) to caproic (C_6:0_) fatty acid.^14^ It is worth noticing that a TAG is considered to be asymmetrical when it has a solitary fatty acid at the *sn*-1 or *sn*-3 position with an acyl chain length differing more than two carbon atoms as compared to the other two fatty acids.^27^

In its free fatty acid form, butyric acid (IUPAC name: butanoic acid)^28^ is volatile and thus contributes to specific sensorial properties of dairy products. It is among the key odorants in cream, and it is described as having a cheese-like aroma.^29^ However, the presence of butyric acid in milk fat is mainly in the form of a TAG. In a recent study, we found that butyryl-containing TAGs account for 13 out of 37 molecules identified in milk fat,^14^ whereas another study reported that this fatty acid appears in 14 out of 56 identified TAGs.^30^ Moreover, Gresti et al.^1^ reported that the three major TAGs in milk fat are all butyryl-containing TAGs, namely, butyryl-palmitoyl-oleoyl-glycerol, butyryl-dipalmitoyl-glycerol, and butyryl-myristoyl-palmytoyl-glycerol. Thus, butyryl-containing asymmetrical TAGs represent quite a significant portion of the milk fat composition. However, despite its high proportion, little information is available on how this group of asymmetrical TAGs contributes to the overall phase behavior or to the polymorphism of milk fat. The asymmetrical TAGs cannot generally form the most stable triclinic β-crystal in a mixture with other symmetric TAGs due to the severe packing constraints.^27^ Therefore, researchers commonly link a high amount of asymmetrical TAGs to the lack of β-polymorphs observed in milk fat.^15^

Among many short-chain butyric-containing TAGs in milk fat, 1-butyryl 2-palmitoyl 3-stearoyl-glycerol (BuPS) is present in concentrations of 3.9 and 4.6% (w/w) in cow milk fat and buffalo milk fat, respectively.^14^ We note that this percentage also accounts for possible positional isomers, for instance, 1-butyryl 2-stearoyl 3-palmitoyl-glycerol (BuSP). However, it can be expected that, due to the small length difference of the palmitic (C_16:0_) and stearic acid (C_18:0_) chains, positional isomers (*sn*-2 and *sn*-3) have little effect on the overall structural arrangement in the crystals. On that account, the present study on BuSP is greatly representative of the general behavior of asymmetrical TAGs in milk fat. Indeed, this work aims to provide an in-depth evaluation of the polymorphism of the butyric-containing asymmetrical TAGs in milk fat and thus provides insights into their role in the bigger scheme of complex milk fat polymorphism. This is achieved by a crystallographic investigation of a pure butyryl-containing TAG compound, applying small- and wide-angle X-ray scattering as well as differential scanning calorimetry. Further, the mechanism of the structural rearrangement, i.e., from prenucleation clustering to tilt angle modification of different crystal polymorphic types, is evaluated by their electron density profiles.

## Material and Methods

### Materials

The 1-butyryl 2-stearoyl 3-palmitoyl-*rac*-glycerol sample (CAS number 152914-63-1) was obtained from Cayman Chemical (Michigan) in the form of a powder with a purity of ≥98%. The sample was received in a temperature-controlled package and then immediately stored in a freezer (*ca*. −18 °C) until the day of experiment.

### Small- and Wide-Angle X-ray Scattering Measurements

The powder sample was directly filled into a 2 mm outer-diameter (1.56 mm inner diameter) thick capillary (Vitrex, Herlev Denmark) without any further preparation. The capillary was subsequently sealed with two-component epoxy glue and a polycarbonate plug. The crystal structure measurements were conducted at the Diamond-Leeds Small Angle X-ray Scattering (DL-SAXS) Facility, situated at Diamond Light Source Ltd., Didcot, United Kingdom. The SAXS instrument, a Xeuss 3.0 from Xenocs SAS (Grenoble, France), was equipped with a molybdenum source and coupled with an Eiger2 R 1 M detector from Dectris AG (Baden-Dätwill, Switzerland). Measurements were carried out at a sample-to-detector distance of 275 mm, resulting in a *q* (4π sin θ/λ) range of 0.08–2.2 Å^–1^, which covered the regime of interest. A Peltier sample stage (Xenocs SAS, Grenoble France) was used to control the sample temperature with an accuracy of 0.1 °C.

Acquisition of the sample scattering patterns of the original powder was acquired at 20 °C for 30 min. Subsequently, the powder was melted at 60 °C and held at this temperature for 15 min. The diffraction patterns of the molten samples were acquired at the same temperature for 15 min. The sample was then quenched to 20 °C and underwent an isothermal hold for 18 h. X-ray scattering patterns were recorded every 15 min during the holding time. The data reduction was carried out in the DAWN 2.24 program^31^ (this concerns (i) the calibration of the *q*-axis, (ii) the radial integration of the two-dimensional (2D)-intensity images, and (iii) the background subtraction of the empty capillary). Further, the data analysis and presentation of data were carried out with Origin 2019b (OriginLab, Massachusetts). For peak fittings, Pearson VII distributions were used. The *d*-spacings were obtained from linear interpolation of all recorded peak positions (*d*(*h*) = 2π/*q*(*h*)).

### Chain Tilt Angle Estimations

**Figure 1:**
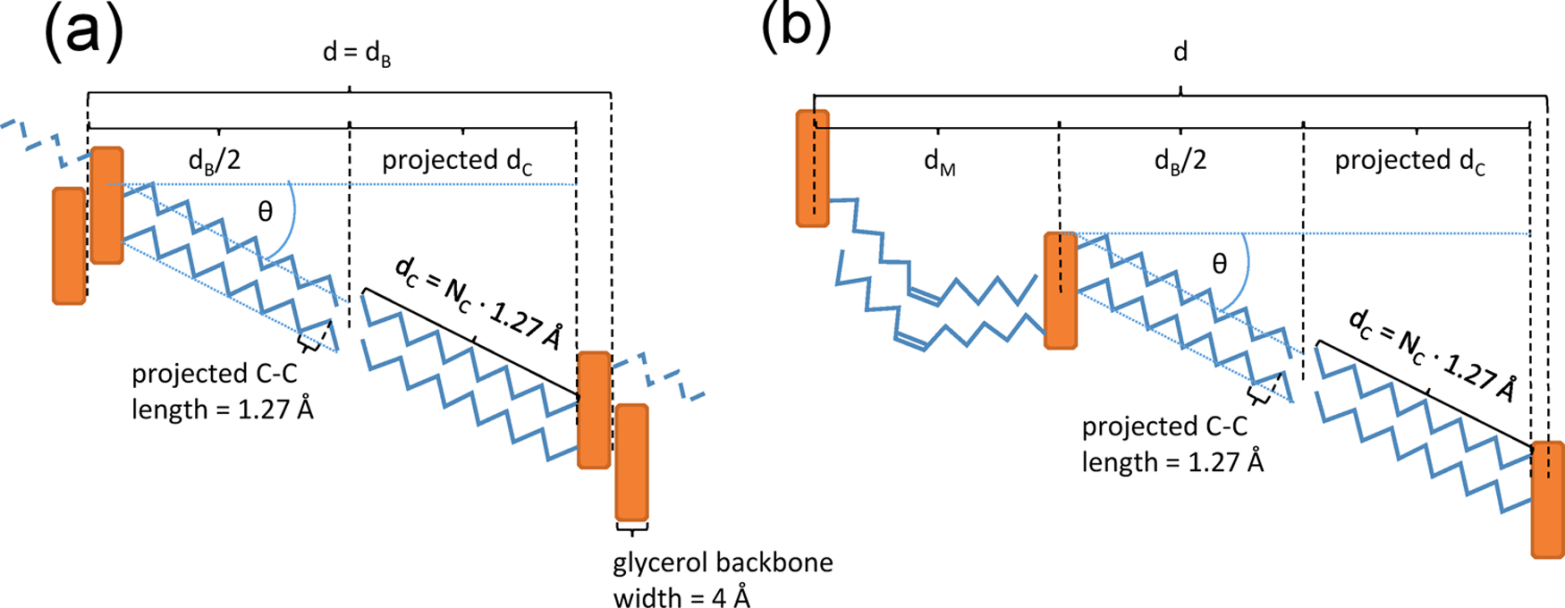
Tilt angle estimation given for 2L-polymorphs (a) and 3L-polymoprhs (b). Hydrocarbon chains are color-coded in blue and the glycerol backbones in orange. The overall thickness in 2L-layers is given by the bilayer thickness (*d*_B_), while in 3L-layers, it is given by the sum of the bilayer and monolayer thickness (*d* = *d*_B_ + *d*_M_).

The given estimations for the tilt angle of the hydrocarbon chains in the 2L- and 3L-phases of TAGs are based on two experimental findings. First, the projected bond length (C–C) along the hydrocarbon chains is known to be 1.27 Å^32^ and the extension of the 1 glycerol backbone in the stacking direction is about 4 Å^33^ (Figure 1). With these estimations, the chain tilt angle in 2L-phases can be expressed as
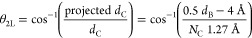
1

Similarly, for 3L-phases, it can be represented as
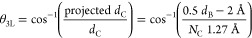
2

It is worth noticing that the estimate for the longitudinal glycerol backbone extension was drawn from data on cocoa butter in the 2L-α phase, and depending on the glycerol backbone orientation in other TAG phases, this value might easily be off by 0.5 to 1 Å.

### Calculation of the Area per Chain

The calculation of the area per chain, *A*_C_, is given by the hexagonal subcell in the α-phase and by the orthorhombic subcell in the β′-phase. In the hexagonal packing, the area per chain is given by
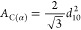
3where *d*_10_ is the *d*-spacing of the only diffraction peak recorded at about *q*_10_ = 1.53 Å^–1^. The area per lipid chain in the β′-phase given by^34^
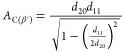
4where *d*_20_ is the corresponding *d*-spacing for the medium-intense peak at about *q*_20_ = 1.64 Å^–1^ and *d*_11_ is the corresponding *d*-spacing of the strong peak at about *q*_*11*_ = 1.51 Å^–1^.^35^

### Electron Density Calculation for the Molten Phase

Electron density profiles of the molten phase are employed to evaluate the prenucleation clustering event of the BuSP. The broad peak at the small-angle scattering regime in the molten phase was simulated using an electron density model developed based on Gaussian distributions. Details of the model development have been described elsewhere.^36^ Briefly, the electron density profile comprises three Gaussian distributions: the first one describes the glycerol backbone of self-assembled triglycerides and is positioned at the center of the profile; the second Gaussian distribution is off-centered at either side of the glycerol headgroup region representing the hydrocarbon methylene groups; and the third Gaussian distribution with a lower height is attributed to the loosely attached triglycerides forming a shell around the core of the assembly. The simulated scattering profile could be obtained by squared Fourier transform of the electron density and then applying a Lorentzian correction. The positions, broadness, as well as height of Gaussian distributions could be optimized by comparing the simulated and experimental scattering profiles. We have applied a particle swarm optimization approach to obtain the best fitting parameters in our model.

### Electron Density Profiles (EDPs)

EDP determinations of BuSP α- and β′-polymorphs were carried out using a standard Fourier transform procedure.^33,37^ Concisely, the Braggs peak intensities were obtained from the fitted area, which were then Lorentz-corrected. The Lorentzian correction of the recorded intensities, *I*(*h*), for a point focus setup, as used in this study, is *h*^2^, where *h* is the Miller index (diffraction order), i.e., all fitted intensities (*I*_h_*)* were multiplied with *h*^2^ (for further reading on the Lorentz correction, see Li et al.^37^). Subsequently, the amplitude values (*F*_h_) were obtained from the square root of the corrected intensities (√*I*_h_*h*). In the case of centrosymmetric EDPs, the Fourier transform is obtained by the summation of cosine terms only
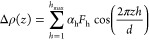
5where Δ*ρ* is the electron density contrast, α_h_ denotes the phases (note, α_h_ is fixed to −1 for *h* = 1), and *d* denotes the lattice spacing. The phases α_h_ for *h* = 2–6 were taken from the literature to be −1, +1, −1, −1, and −1.^38^

### Differential Scanning Calorimetry (DSC) Measurements

Heat flow measurements were carried out with a TA instrument DSC Q-20 (Elstree, U.K.). An 8 ± 0.01 mg BuSP powder sample was readily inserted into an aluminum T-zero pan (TA instruments, Elstree, U.K.) and hermetically sealed. The heat flow of the sample was calculated as the net heat flow difference between the sample-containing pan and an empty reference pan.

The applied thermal procedure consisted of three subsequent heating and cooling ramps. The heating rate was 5 °C/min, whereas the cooling rate was −10 °C/min for the entire experiment. First, the sample powder was equilibrated at 20 °C before being heated to 65 °C. The sample was held at this temperature for 2 min before being subjected to a cooling back to 20 °C. Afterward, the temperature was kept constant for 1 h followed by a heating ramp to 65 °C to remelt the sample. Similarly, a 2 min hold was applied before the sample was brought to 20 °C. Last, an isothermal hold for 18 h was carried out before the final melting ramp to 65 °C. All experiments were conducted in triplicate. Data were processed using TA Instruments Universal Analysis 2000 version 4.5A and plotted in Origin 2019b (OriginLab, Massachusetts).

## Results and Discussion

### Crystallization Kinetics during Isothermal Hold

**Figure 2:**
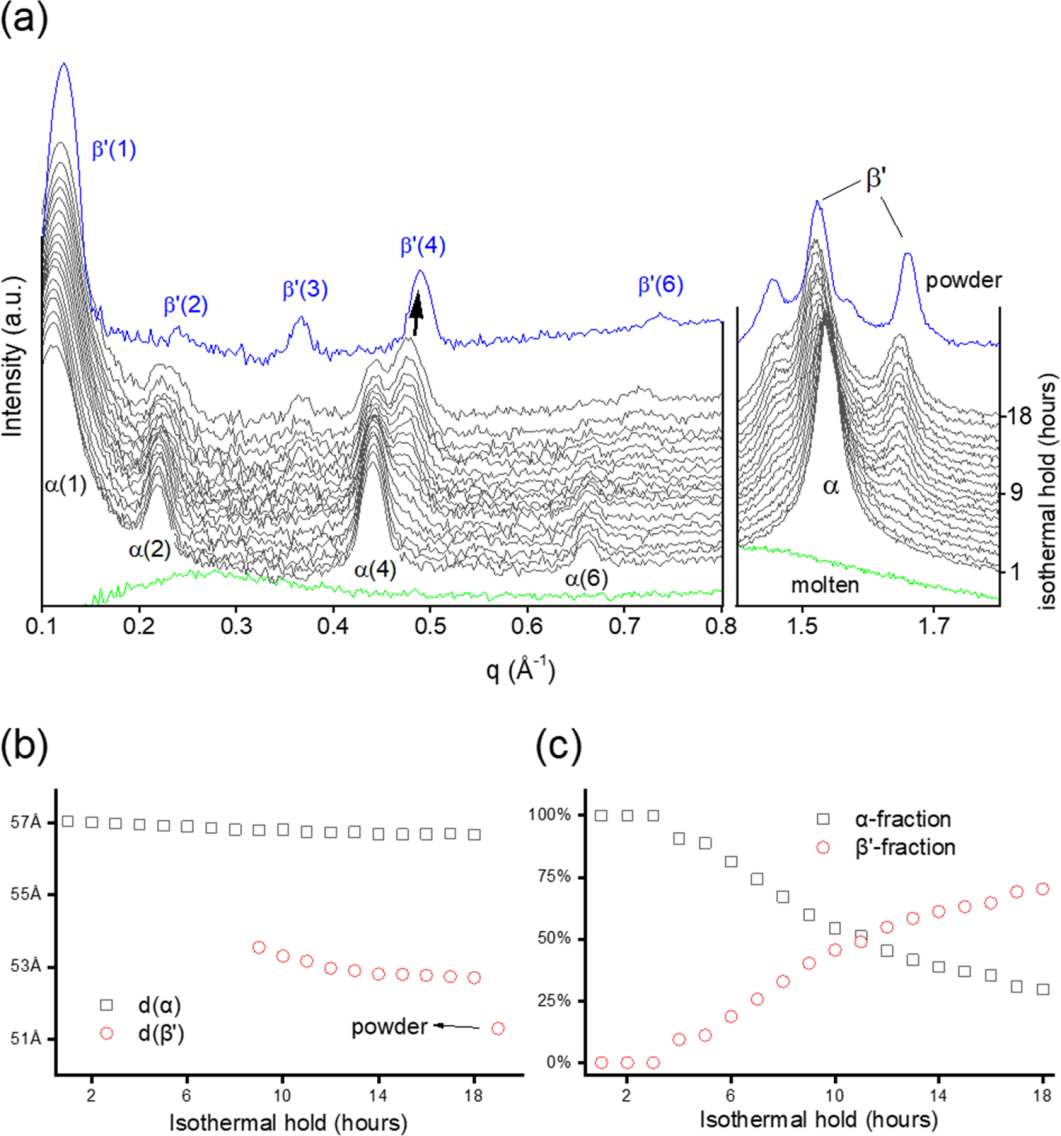
(a) Evolution of the BuSP structure at 20 °C during an isothermal hold for 18 h as observed by small- and wide-angle X-ray scattering. Note, for each nonzero diffraction peak, the corresponding Miller index is given in brackets. (b) *d*-spacing of the α- and β′-polymorphs. (c) Estimated crystalline fractions of the α- and β′-polymorphs over the isothermal hold time.

The temporal evolution of BuSP X-ray diffraction patterns during the isothermal hold at 20 °C is presented in Figure 2a. The crystallization from the molten BuSP occurred immediately after the isothermal target temperature was reached. A crystal structure corresponding to a hexagonal packing was observed during the first hour of isothermal hold, with a stacking distance of 57 Å. On further observation with a longer isothermal hold, a polymorphic transformation from the metastable α- to the more stable β′-polymorph took place. Most notable indications are the decaying intensities of the fourth- and sixth-order peaks of the α-phase, while all of the β′-peaks are rising in intensity, as clearly noticeable by its fourth-order peak at *q* = 0.48 Å^–1^. The polymorph transformation can be observed also in the wide-angle region, where the short spacing peak at *q*_20_ = 1.64 Å^–1^ (*d*_20_ = 3.84 Å) grows in intensity. Note, together with the *q*_11_-peak at 1.51 Å^–1^ (*d*_11_ = 4.15 Å), the *q*_20_ peak defines the orthorhombic subcell of the β′-polymorph chain packing^35^ (eq 4).

Figure 2b shows the time evolution of the lamellar repeat distances of both the α- and β′-polymorphs. Note that in the coexistence regime, these stacking distances were estimated from the relatively strong and well-resolved fourth-order peaks. The strongest first-order peak positions were not considered in this analysis as they are widely overlapping. The lamellar thickness of the α-form was observed to decrease only slightly from 57.0 to 56.7 Å at the end of the isothermal hold. The β′-polymorph peaks were observed first after 3 h of the isothermal hold (see Figure 2a, WAXS). However, the first reliable fourth-order peak in the SAXS region could only be clearly obtained from the 9^th^ hour onwards, which displayed a lamellar thickness of 53.5 Å. The *d*-spacing of the β′-form decreased to 52.7 Å at the end of the isothermal hold. Further comparison of this value with the X-ray diffraction of the powder sample shows that the β′-crystal eventually evolves to an even more compact structure, with a *d*-spacing of 51.2 Å.

Figure 2c shows a binary transformation from the α- to the β′-polymorph. To estimate the polymorph fractions as a function of time, we used the intensity of the *q*_20_ peak of the orthorhombic subcell (see Figure 2a, WAXS). The highest intensity of the *q*_20_ peak from the powder sample was set as 100% of the β′-fraction, whereas the pure α-system at isothermal *t* = 1 h represents 100% of the α-fraction. As a result, at the end of the isothermal hold (18 h), we estimate that the BuSP crystalline material consisted of around 70% β′-form and 30% α-form. Additional conversions of molten TAGs to the crystalline phases have not been considered. Indeed, the α- to β′- polymorphic conversion is accompanied by a continuous increase in the solid fat content (SFC). Estimations from the wide-angle X-ray patterns^39^ show that the SFC increases from 47 to 76% from *t* = 1 to *t* = 18 h of isothermal hold. Details on SFC estimations are presented in the Supporting Information Figure S1.

The kinetics of the BuSP α- to β′-crystal transformation is slow if we compare it with that of milk fat. In our previous study, the α-polymorph in milk fat had already disappeared after 5 h of isothermal hold at −10 °C.^14^ Note, in this study, we employ a higher isothermal hold temperature (20 °C), which should promote an even faster polymorph transformation toward the β′-crystal,^15^ as this temperature is closer to the melting point of the α-phase. It was also reported that milk fat can directly crystallize into the β′-form after quenching to 25 °C,^40^ which demonstrates its favorable pathway to stabilize in the orthorhombic packing of the hydrocarbon chains. Hence, the slower temporal evolution of BuSP in this study supports the notion that asymmetric TAGs may play a significant role in delaying the polymorphic transformation from α- to β′-crystal in milk fat. We note that this view is also in agreement with our previous findings,^14^ where buffalo milk fat, which has higher butyryl-containing TAGs than cow milk fat, exhibits slower α to β′ polymorph transformation at a cooling rate of −0.5 °C/min.

### Polymorphism of BuSP

**Figure 3:**
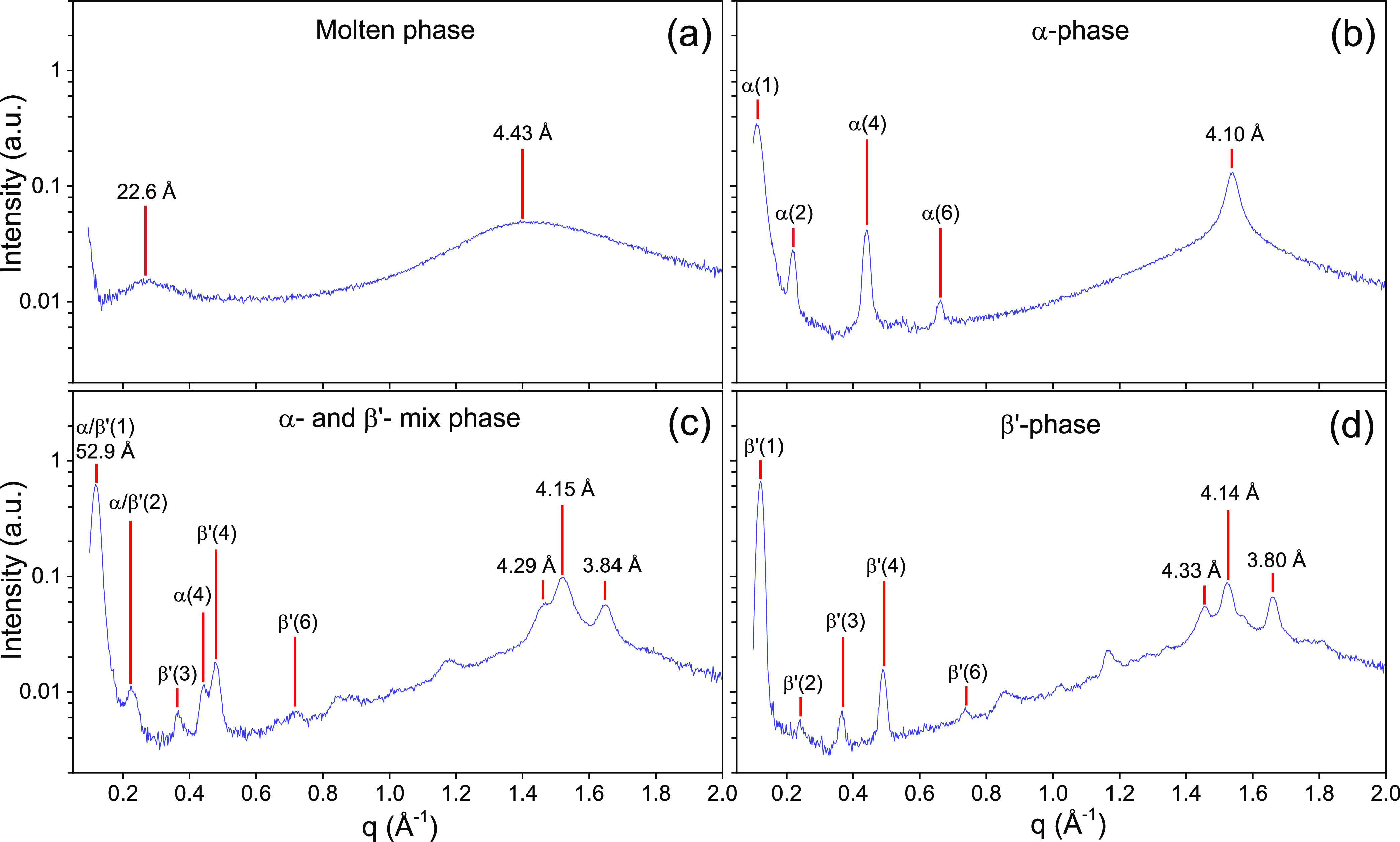
X-ray diffraction patterns of 1-butyryl 2-stearoyl 3-palmitoyl-glycerol at different phases: (a) molten, sample heated at 60 °C for 15 min; (b) α-phase, molten sample quenched to 20 °C and held for 1 h; (c) mix of α- and β′-phases, molten sample quenched to 20 °C and held for 18 h, and (d) β′-phase, direct observation on the powder sample.

**Figure 4:**
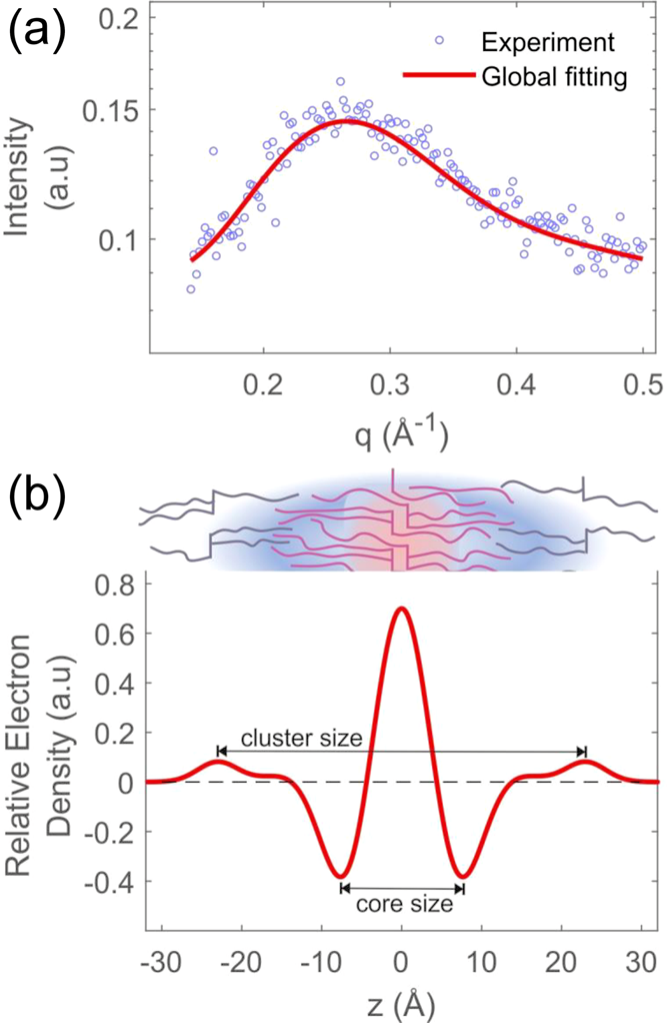
(a) Global fitting (line) of the SAXS pattern from BuSP in the molten phase (open circles) based on the structural TAG cluster model presented below. (b) Electron density profile demonstrating the BuSP assembly in a core–shell cluster model. The outmost peaks represent loosely attached BuSPs to the cluster core.

For obtaining a better understanding of the nanostructure of the different polymorphs of BuSP, selected X-ray diffraction patterns are presented in more detail in Figure 3. For BuSP heated to 60 °C, Figure 3a shows two broad scattering peaks corresponding to characteristic lengths of 22.6 and 4.43 Å, respectively. The first diffuse peak arises from the clustering of molten TAG molecules^33^ and the second diffuse peak stems from the short-range interchain correlation peak position.^41^ Concerning the clustering behavior, we have recently presented a “core–shell cluster” model to elucidate this phenomenon.^36^ The model describes the TAGs being arranged in a “back-to-back” fashion in its core, while an outer layer of TAGs is only loosely attached to it. Employing the model, we have fitted the molten BuSP scattering patterns with this three-Gaussian model,^36^ obtaining a very similar cluster model (Figure 4) as determined for cocoa butter (CB).^36^

Briefly, the overall BuSP cluster size (Figure 4b) is 46 Å, leading to a fluid “bilayer” thickness of 23 Å, which compares to a determined chain length of palmitic and stearic acids of 13–14 Å.^42^ Note, the term “bilayer” is not used strictly here. First, our model cannot pinpoint whether the TAG clusters have a planar or rodlike geometry, and further, the second loosely attached TAG-layer coverage is only about 8%. Further, considering the extension of the glycerol backbones to occupy about 6 Å per “bilayer” (a full glycerol backbone extension in the core, 4 Å, and half at the outer layer, 2 Å), we can conclude that the core and second-layer interdigitate up to about 9 Å. At this stage, it remains speculative, but this observed interdigitation might be caused by fatty acyls not only orienting outward from the center of the core but also reaching into the opposite side of the cluster and hence creating a void deeper inside the core, which gets occupied by the second-layer TAGs. This would apply for TAGs being in the tuning fork (Tf) and chair conformation (Ch), while the trident (Tr) TAGs would not cause any interdigitation effect in this picture. As already assumed in our original paper on TAG cluster formation,^36^ the propeller (Pr) TAGs would fit best into the clusters at the edges, i.e., into the highly curved areas of the TAG cluster. In this respect, it is instructive to know that the probability of finding a TAG in a specific conformation in the fluid phase was recently simulated to follow the order of Tr (50–56%) > Ch (28–34%) > Pr (11–14%) > Tf (1–6%),^43^ allowing for the proposed TAG cluster formation. Finally, since the BuSP and CB^36^ cluster analyses showed practically identical structural extensions, we do believe that butyric acid plays only a minor role in fluid cluster formation.

Returning to our overview graph, the X-ray scattering pattern of the BuSP α-polymorph is shown in Figure 3b. The single peak at wide angle with a short spacing of 4.10 Å corresponds to a hexagonal lateral packing of the hydrocarbon chains, whereas in the small-angle regime, diffraction peaks covering the first six orders arise from the 3L stacking structure with a *d*-spacing of 56.9 Å. Here, the calculated *d*-spacing accuracy is slightly improved from the incorporation of all peaks, as compared to Figure 2b, where only the fourth-order peak was considered.

Figure 3c shows the diffraction pattern of BuSP displaying the coexistence of the α- and β′-polymorphs, which was taken after 18 h of isothermal hold. The presence of the β′-form is clearly indicated by the *q*_20_ peak at 2π/3.84 Å^–1^. At this specific isothermal time, the α-polymorph decreased significantly but did not entirely vanish. This can be seen from the less-intense fourth-order peak (*q* = 0.441 Å^–1^) of the α-phase, which coexists with the more intense fourth-order peak (*q* = 0.477 Å^–1^) of the β′-phase. Noteworthy, the position of the overlapping first-order peaks corresponds to 52.9 Å. This long spacing is very close to the *d*-spacing of the ambiguous 3L structure (53 Å) previously observed in milk fat.^14,21^ Thus, it is tempting to conclude that this 53 Å spacing most likely stems from the mixed α- and β′-phases of butyryl-containing TAGs. This conjecture is also in accordance with the fact that BuSP exhibits relatively slow polymorphic transformation kinetics compared to milk fat in general.

The X-ray scattering pattern of the pure β′-polymorph of BuSP was obtained from the powder sample (Figure 3d). Note, no traces of the α-polymorph were detected here. Again, we were able to record the first six-orders in the small-angle regime, with a nonzero third-order peak being unique as it was not recorded in the α-form (Figure 3b). Taking all peaks into consideration, the longitudinal *d*-spacing is 51.2 Å. In the wide-angle regime, three dominant peaks are apparent, i.e., referring to the short spacings of 4.33, 4.14, and 3.80 Å, with the second peak having the strongest intensity. In agreement with the literature,^38,44^ this diffraction pattern is attributed to the form IV of the β′ polymorph in cocoa butter. We note, while the characteristic three diffraction peak positions vary only little among different form IV polymorph structures,^44^ the intensity profiles do display differences. For instance, for cocoa butter samples, the 4.33- and 4.14-related peaks are the most intense, while other samples such SOS- and POP-rich samples display a similar intensity profile as BuSP.^38^ Using eqs 3 and 4, we calculated the area per chain, *A*_C_, in the pure α phase and the pure β′ phase. Clearly, *A*_C_ decays from 19.4 to 18.8 Å^2^, which is in good agreement with other reported hydrocarbon chain packings.^45,46^

We note that our BuSP powder sample was received in a temperature-controlled package, and it was then kept in the freezer for more than one month before measurement. Therefore, we argue that the sample should be the most stable crystal form of this asymmetrical TAG. On that note, the current results show that our previous hypothesis^14^ on asymmetrical TAGs being linked to a β-polymorph appearance in milk fat can no longer be upheld. An intense peak corresponding to the short spacing of 4.6 Å is the main indication for a triclinic chain packing as found in the β-polymorph.^47^ However, this peak was not observed in our experiments (Figure 3d). BuSP instead seems to be stable in an orthorhombic packing configuration and is unlikely to transform into a triclinic-based structure.

**Figure 5:**
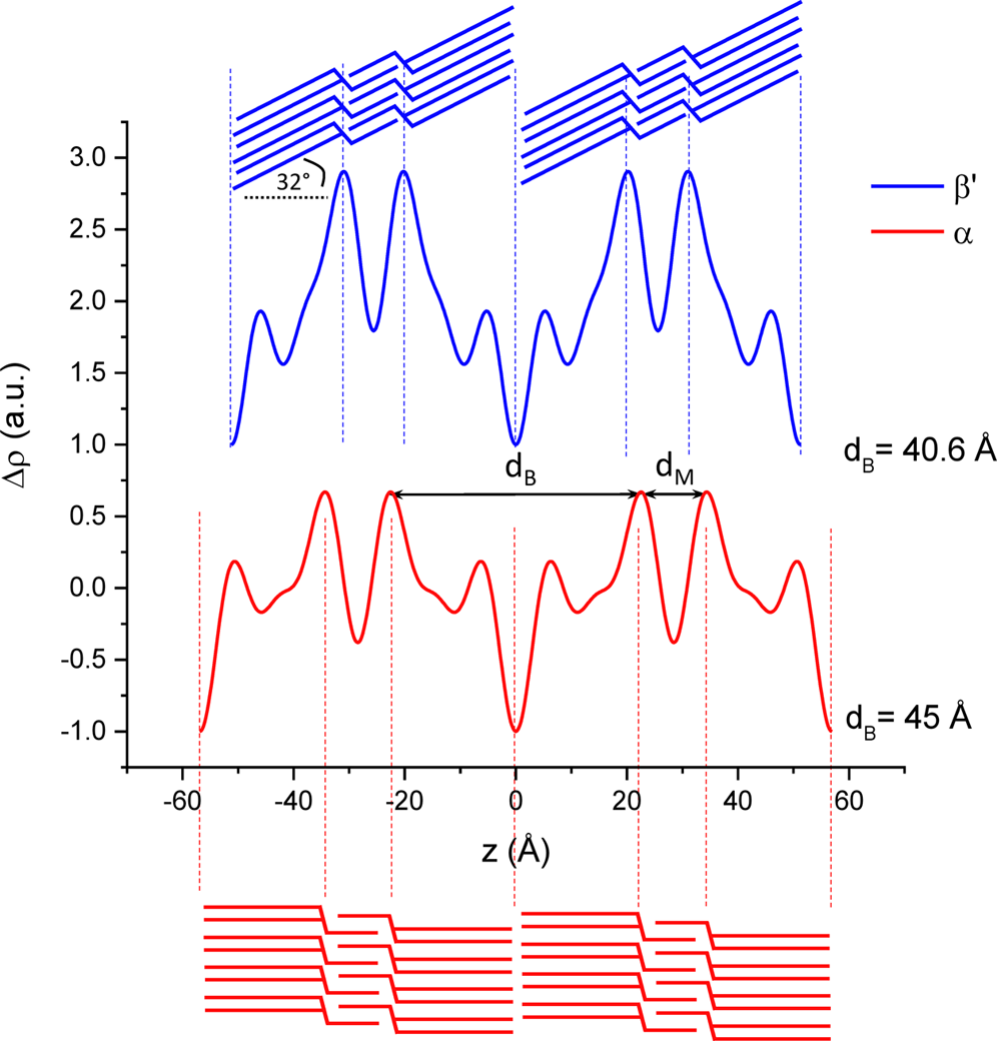
Electron density profiles (EDPs) of 1-butyryl 2-palmitoyl 3-stearoyl-glycerol (BuSP) determined from X-ray diffraction patterns presented in Figure 2. The α-polymorph has a *d*-spacing of 56.9 Å (*d*_B_ = 45 Å), and the β′-polymorph has a *d*-spacing of 51.2 Å (*d*_B_ = 40.6 Å). *d*_B_, bilayer thickness; *d*_M_, monolayer thickness.

**Figure 6:**
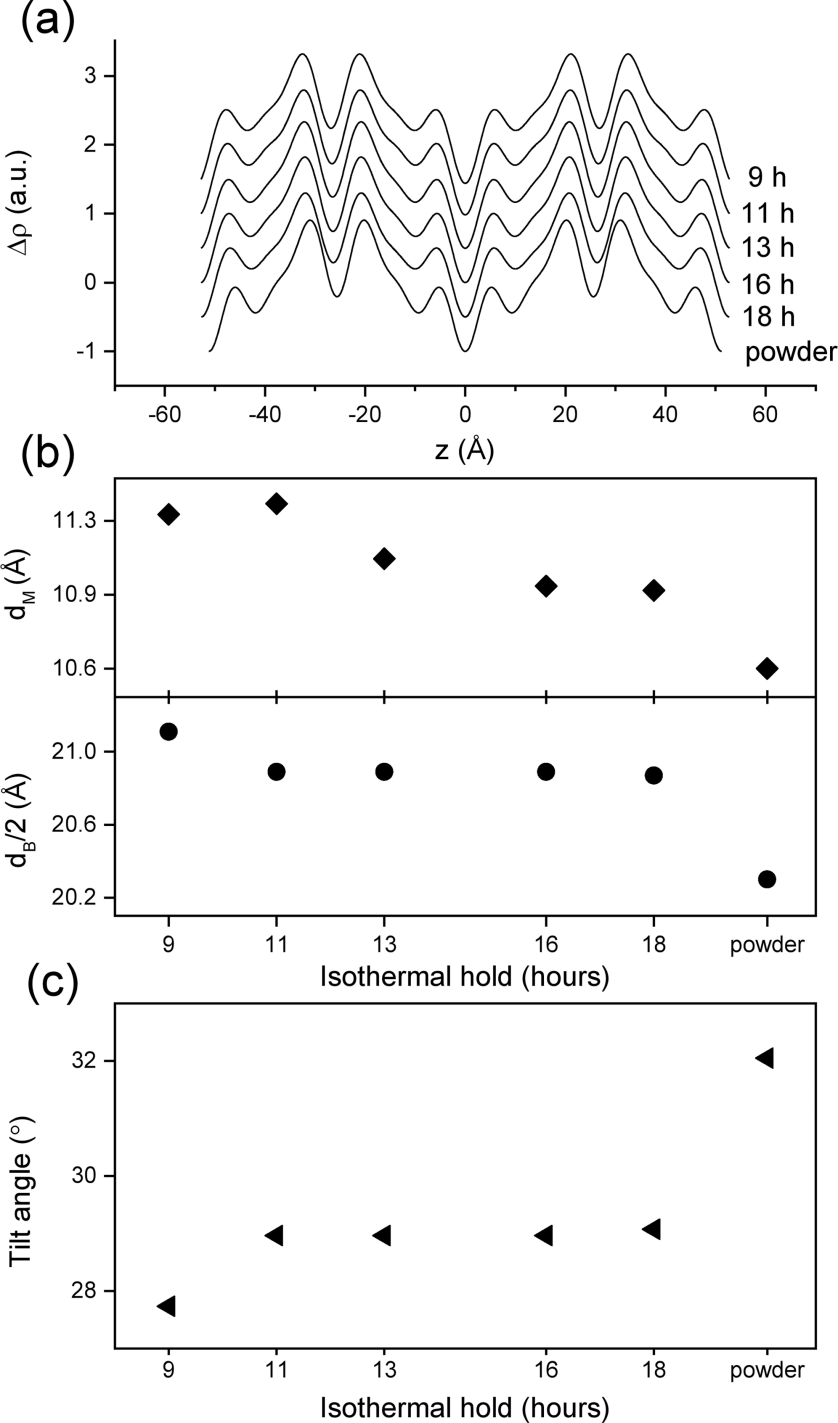
(a) Evolution of electron density profiles (EDPs) of 1-butyryl 2-palmitoyl 3-stearoyl-glycerol (BuSP) β′-polymorph at different isothermal holds. (b) Evolution of bilayer and monolayer thickness of the BuSP β′-polymorph at different isothermal holds. (c) Evolution of the tilt angle of the BuSP β′-polymorph at different isothermal holds.

The inhibition of the β-polymorph formation in BuSP may stem from the density of butyryl chain packing in the monolayer regime being too low as compared to a common oleic acid monolayer arrangement, for instance, in stearoyl-oleoyl-stearoyl-glycerol (SOS). Indeed, the EDPs of BuSP (Figure 5) do display relatively lower densities when compared to the monolayer regime of SOS in the stable β phase.^37^ As detailed in Figure 6b, we estimate a loose interdigitation of the butyryl chain in the monolayer regime causing reduced van der Waals forces, which in turn does not allow a denser packing in the bilayer region (β-polymorph). This evaluation is in accordance with the earlier work of Kodali et al.,^48^ who studied the polymorphism of 1,2-dipalmitoyl-3-acyl-*sn*-glycerols, with the *sn*-3-acyl substituted by 2–16 even-numbered saturated fatty acyl chains (PP2–PP16). The current results show similar behavior with 1,2-dipalmitoyl-3-hexanoyl-glycerols (PP6), where the compound is the most stable in the β′-form with a 3L lamellar architecture, but not with PP4 (reported 2L-stacking). We speculate the difference in BuSP may arise from methyl end stacking interaction, i.e., stearoyl-palmitoyl *vs* palmitoyl-palmitoyl, which influences the chain-length organization.^27^ Remarkably, Goto et al.^49^ observed a 3L stacking even in PP2 (single crystals), which agrees with the current finding. However, it is worth noting that the currently observed behavior of BuSP is from a racemic compound, whereas the optically active version of the BuSP might show a different crystallization behavior, as seen in other asymmetrical triacylglycerols.^50^ Nevertheless, this work provides further support to the view that the asymmetrical TAGs are typically stable in the β′-polymorph.^27^ Consequently, the high proportion of these TAGs in milk fat could be a contributing factor to the β′-polymorph being predominant in milk fat crystals.^15^ A summary of the BuSP α- and β′-polymorph X-ray crystallographic parameters is presented in Table 1.

**Table 1:** Summary of *d*-Spacing, Small-Angle Peak Intensity, and Short Spacings of BuSP Polymorphs^a^

		intensity of small-angle peaks								
polymorph	*d*-spacing (Å)	1	2	3	4	5	6	short spacings (Å)		
α	56.9	vs	w		m		vw		4.10 (vs)	
β′	51.2	vs	vw	w	m		vw	4.33 (m)	4.14 (s)	3.80 (s)

a vs, very strong; s, strong; m, medium; w, weak; vw, very weak.

### Electron Density Profiles (EDPs) and Structural Evolution of the β′-Polymorph

EDP is a useful tool to further obtain fine-structural information from X-ray scattering data.^37^ For instance, the maxima in the EDPs represent the highest electron density, which in the case of TAGs is given by the carbonyl groups in the glycerol backbone. Accordingly, one can utilize the peak-to-peak distance in the EDPs to obtain the inner structure of the TAG lamellae, such as the bilayer and monolayer thickness. Further, by knowing the bilayer thickness, one can work out the tilt angle and estimate the number of its contributing carbons in the chain^14^ (see Figure 1 as well as eqs 1 and 2).

Figure 5 shows the EDPs of α- and β′-polymorphs of BuSP, whose structural parameters are given in Table 2. It is clearly displayed that the BuSP crystals are organized as 3L architectures, as shown by two maxima corresponding to two separated glycerol backbone regions. By contrast, a 2L structure would only manifest a single maximum per unit cell stemming from the back-to-back arrangements of glycerol backbones. The shorter distance between the maxima in Figure 5 indicates that the monolayer region can only be occupied by the short-chain butyryl, whereas the longer stearoyl and palmitoyl chains aggregate in the bilayer region. It is worth noticing that all three alkyl chains of BuSP are saturated fatty acids; thus, we assume all three to be in their all-trans conformation. The EDPs are constructed from four and five nonzero diffraction peaks for the α- and β′-polymorphs, respectively. Nevertheless, we recorded in both cases diffraction peaks up to the sixth Bragg’s order. While the resolution of the EDPs should scale roughly with half of the smallest recorded lattice spacing (i.e., about 5 Å), the precision finding the maxima in the EDP at this given resolution is expected to be below 1 Å.^51^ The bilayer thickness determined from the β′-polymorph EDP (Figure 5) is 40.6 Å, while the number of contributing carbons for BuSP is taken as an average of that of stearic and palmitic acids (*N*_C_ = 17). The β′-form tilt angle for a 3L structure was calculated using eq 2 as introduced in the Materials and Methods section. Hence, the calculated tilt angle of the BuSP β′-polymorph is about 32°. The previously reported chain tilt for the most stable β-form of stearoyl-oleoyl-stearoyl-glycerol was 36°.^38^ Therefore, the current result is in excellent agreement with the common knowledge that the β′ structure has a looser chain packing than the β one, and consequently, it displays a smaller tilt angle.

**Table 2:** Structural Parameters Used for the Calculation of the BuSP Electron Density Profiles

	α-polymorph			β′-polymorph		
h	*q* (Å^–1^)	*I*_h_ (a.u.)	α_h_ *F*_h_ (a.u.)	*q* (Å^–1^)	*I*_h_ (a.u.)	α_h_ *F*_h_ (a.u.)
1	0.1104	1.000	–1.00	0.1219	1.000	–1.00
2	0.2189	0.042	–0.41	0.2454	0.004	–0.12
3			+0.00	0.3660	0.008	+0.27
4	0.4405	0.090	–1.20	0.4899	0.026	–0.64
5			–0.00			–0.00
6	0.6619	0.011	–0.62	0.7368	0.004	–0.36

The hexagonally packed α-polymorph chains are freely rotating around their long axis; thus, the chain packing tilt is expected to be about 0°. This is further confirmed by the EDP-deduced chain length *d*_C_, which we estimated to be equal to *d*_B_/2–2 Å (see Figure 1), i.e., 45/2–2 Å = 20.5 Å. This compares well to an all-trans chain with 17 carbons: *d*_C_ = 17 · 1.27 Å = 21.6 Å. Note that the deviation is within the errors and might be caused by both the given uncertainty on the longitudinal glycerol backbone extension of 4 Å and the positional error of the EDP maxima.

Chain packing tilt is a consequence of the conformational rearrangement of the TAG molecules toward a more compact structure during a polymorphic transformation. In this respect, one observes for instance an increasing chain tilt from the metastable β′ to the stable β polymorph. However, a tilt angle evolution is also observed during finer structural rearrangements within a phase, for instance, during the condensation process from initial β′-polymorph formation toward the final and stable organization of this packing, as already indicated by the shrinking of its *d*-spacing (Figure 2b). Figure 6a,c displays a series of EDPs and the corresponding tilt angle evolution of the BuSP β′-crystal as a function of elapsed time.

It is worth noticing that the determination of EDPs, when the α- and β′-crystals coexist (Figure 3c), required an intensity correction procedure because of the strongly overlapping first- and second-order peaks. For this reason, the fitted areas of both peaks were corrected by the corresponding fraction of the β′-polymorph (Figure 2c). In conclusion, we can associate the shortening *d*-spacing of the β′-form (Figure 2b) with an increasing chain packing tilt (Figure 6c). At *t* = 9 h, the β′-crystal is observed with an estimated chain tilt of 27.7° (Figure 6c). The crystal becomes more compact, and its lamellae condense with a longer isothermal hold. At the end of the experiment (*t* = 18 h), the tilt angle increases to 29.1°. Taking the BuSP powder as the final β′-polymorph architecture, we can infer that this packing readjustment will continue until the final chain tilt of 32° is reached.

Figure 6b shows the evolution of the bilayer and monolayer thickness of the β′-form as a function of time. It is worth noting that not only the bilayer but also the monolayer thickness decreases during the longer isothermal hold. This shows that the more compact β′-stacking (Figure 6b) not only is reflected in increasing chain tilts but is also due to changes in the monolayer region. In fact, the monolayer thickness, *d*_*M*_, changes from 11.3, 11.0, to 10.6 Å for *t* = 9 h, *t* = 18 h, and *t* > 30 days, respectively. A plausible explanation for this would be that the degree of interdigitation augments with time in this region, and hence, it also contributes to the condensing of the β′ polymorph. Indeed, the estimated all-trans chains length, *d*_C_, of butyryl is only 5.1 Å (*N*_C_ · 1.27 Å = 4 · 1.27 Å), while the monolayer region accommodates spaces of 6.6–7.3 Å (*d_M_* – 4 Å). Nevertheless, even though the trends in *d*_M_ are significant, absolute errors 0.5–1.0 Å still have to be considered.

### Thermal Characterization of BuSP

**Figure 7:**
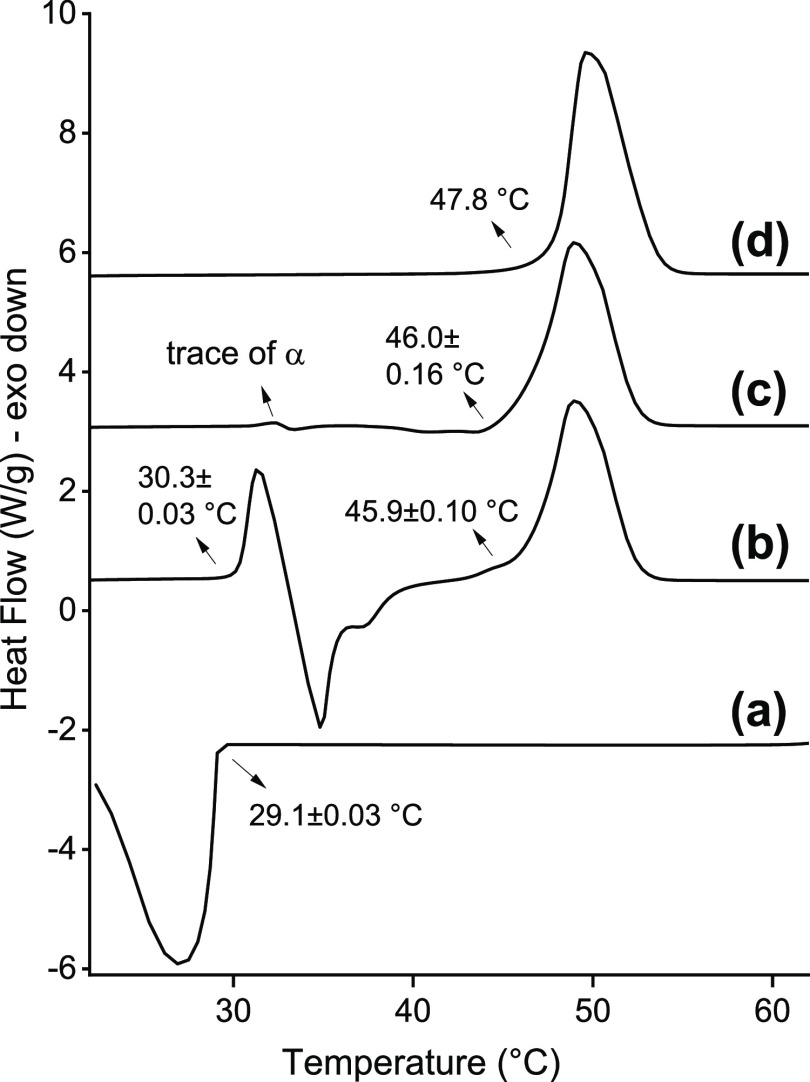
Thermogram of BuSP at different thermal conditions: (a) melt cooling from 65 to 20° at 10 °C/min rate, (b) the molten sample is equilibrated at 20 °C for 1 h before being heated to 65 °C at 5 °C/min rate, (c) the molten sample is equilibrated at 20 °C for 18 h before being heated to 65 °C at 5 °C/min rate, and (d) the powder sample is heated to 65 °C at 5 °C/min rate.

Thermograms of BuSP subjected to different thermal treatments are presented in Figure 7. Four experimental setups were carried out to complement the X-ray scattering observation. The first one is to observe the crystallization temperature of BuSP from its melt (Figure 7a). When subjected to a −10°C/min cooling rate, the onset of the exothermic peak shows that the BuSP melt has a nucleation point of 29.1 ± 0.03 °C. As a comparison, cow and buffalo milk fat show nucleation points at 15.6 and 17.4 °C, respectively, at the same cooling rate.^14^ Therefore, BuSP is among the higher melting point TAGs in milk fat, most likely due to its fully saturated chains. Nonetheless, it melts at lower temperatures than fully saturated long-chain TAGs, such as tripalmitin, which reaches 66 °C.^52^

To produce the α-polymorph, the second thermal treatment was an isothermal hold for 1 h at 20 °C following crystallization. Upon heating at a 5 °C/min rate, the thermogram exhibits two endothermic and one exothermic peak (Figure 7b). The first endothermic peak corresponds to the melting of the BuSP α-polymorph, with an onset point of 30.3 ± 0.03 °C. This is followed by an exothermic dip that corresponds to a polymorphic crystallization α → β′ polymorph. Hence, the second endothermic peak could be attributed to the melting of the β′-phase with the onset point at 45.9 ± 0.1 °C.

Figure 7c shows the thermogram of BuSP melting after a long isothermal hold (18 h) as the third thermal treatment. From X-ray measurements (Figure 2), we observed a significant, but not complete, α → β′ polymorphic transformation with this treatment. The thermogram shows incomplete transformation, as indicated by a small hump at about 32 °C, corresponding to traces of α-form melting. In comparison to the 1 h isothermal hold, a deep valley corresponding to an exothermic event (e.g., a polymorphic transformation) is not observable. In turn, one endothermic peak is observed, which corresponds to the β′-polymorph melting. The peak onset is 46.0 ± 0.16 °C, which is quite similar to that of the 1 h isothermal hold. It is interesting to compare both onset points with the last thermal treatment, a direct melting of BuSP powder at the heating rate of 5°C/min (Figure 7d). The powder melting displays an onset point at 47.8 °C, which is about 1.6 °C higher than the isothermally treated samples. Correlating this with the above observations from X-ray scattering data, we can associate the higher melting point as the result of a more stable and compact structure, albeit the same crystal packing architecture of a β′-polymorph is present. It is worth noticing that the powder could be melted only once due to sample limitation.

## Conclusions

The polymorphism of asymmetrical TAGs in milk fat, as represented by BuSP, was studied here for the first time. BuSP shows only α- and β′-polymorphs with *d*-spacings of 56.9 and 51.2 Å, respectively. The most stable β-polymorph is not observed, even after months, possibly due to the relatively lower fatty acyl density in the monolayer region, prohibiting a denser bilayer packing as needed for the formation of the β phase.

BuSP also displays a relatively slower polymorphic transition from the α to β′ phase, when compared to milk fat. The most condensed structure was observed in the pure β′-phase after being kept for more than one month at −18 °C, with a tilt angle of 32° and monolayer thickness of 10.6 Å. Melting point observations using differential scanning calorimetry confirm this most compact structure. Finally, the modeled molten phase structure shows that the butyryl chains do not play a significant role in the clustering of TAGs, but its architecture is mainly dominated by stearic and palmitic fatty acids. The TAGs preferentially distribute in different cluster regions due to their tuning, chair, trident, and propeller conformations.

This specific polymorphism of BuSP gives valuable insights into the crystallization behavior of butyryl-containing TAGs in milk fat and, further, how the behavior of these asymmetrical TAGs plays a role in overall milk fat crystallization. At higher crystallization temperatures, asymmetrical TAGs containing unsaturated fatty acyl chains may likely be part of the liquid fraction due to its lower nucleation point. Moreover, because of its slow polymorphic transformation, it is plausible to predict that a high concentration of asymmetrical TAGs might delay the polymorphic transformation from the α to β′ phase in milk fat crystallization. Lastly, the asymmetrical TAGs do not tend to form the β polymorph, which could be a major factor for the common absence of the β polymorph in milk fat.

## Supporting Information Available

Additional solid fat content (SFC) estimations using the WAXS data, including two figures (PDF)
