# Supplementary material for: Polymorphism of a Highly Asymmetrical Triacylglycerol in Milk Fat: 1-Butyryl 2-Stearoyl 3-Palmitoyl-glycerol
Source: Cryst Growth Des. 2022 Sep 13;22(10):6120–30. doi: 10.1021/acs.cgd.2c00713 (PMC9542709; doi:10.1021/acs.cgd.2c00713)
Supplement: Supplementary file 1 — cg2c00713_si_001.pdf [file cg2c00713_si_001.pdf]

SUPPORTING INFORMATION

Polymorphism of a highly asymmetrical triacylglycerol in milk fat: 1-butyryl 2-stearoyl 3-palmitoyl-glycerol

Yoga Pratama[1,2], Sam Burholt[1,3], Daniel L. Baker[4], Amin Sadeghpour[1], Elena Simone[5], Michael Rappolt[1]*

[1]School of Food Science and Nutrition, Food Colloids and Bioprocessing Group, University of Leeds, Leeds LS2 9JT, United Kingdom

[2]Department of Food Technology, Faculty of Animal and Agricultural Sciences, Diponegoro University, Semarang 50275, Indonesia

[3]Diamond-Leeds Small Angle X-ray Scattering Facility, Didcot Oxfordshire OX11 0DE, United Kingdom

[4]School of Physics and Astronomy, University of Leeds, Leeds, LS2 9JT, United Kingdom

[5]Department of Applied Science and Technology, Politecnico di Torino, Torino 10129, Italy

*E-mail address of the corresponding author: m.rappolt@leeds.ac.uk

Solid Fat Content (SFC) Estimations Using the Wide Angle X-ray Scattering (WAXS) Data

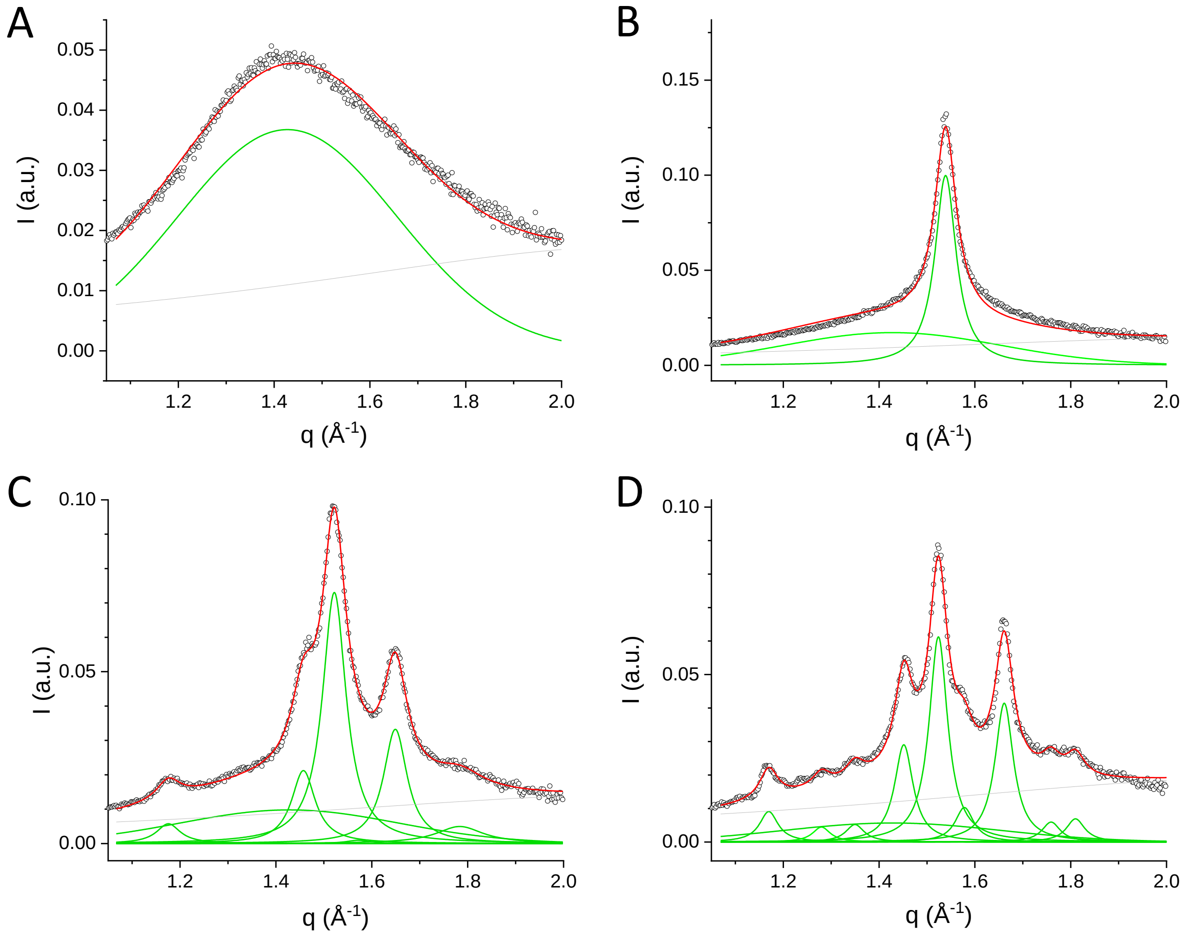

***Figure S1**. SFC estimation using the WAXS data. This analysis does follow the ideas outlined in the reference [1] (A) 1-butyryl 2-stearoyl 3-palmitoyl-glycerol (BuSP) sample at 60 °C before cooling down to 20 °C. Here the **SFC = 0%**. (B) After one hour of isothermal hold at 20 °C the α-phase forms. The **SFC = 46%**. (C) After 18 hours the newly formed β'-phase still coexists with the α-phase. The **SFC = 76%**. The BuSP sample that was stored before X-ray measurements at -18 °C for 720 hours (1 month) displays solely the β'-phase. The **SFC = 80%**. Grey lines indicate background scattering, the green lines display all diffraction peaks as well as the diffuse scattering of the fluid phase. The red lines display the fitted data curves. Note, the background scattering is the same in all patterns, and moreover, the fluid phase diffuse scattering was fixed in its width and position. IMPORTANT NOTE: Applying the method of the Elke Scholten group using Pearson VII functions for all scattering contributions[1], we did fix the shape of the diffuse scattering to a Gaussian shape (m = 100) and that of the diffraction peaks to a Lorentzian shape (m = 1), which is the expected peak shape for smectic liquid crystalline phases. We note, that not fixing the shape of the peaks can lead to artificial results, such as overestimating or underestimating peak areas due to different tailing off behaviour of differently shaped peaks.*

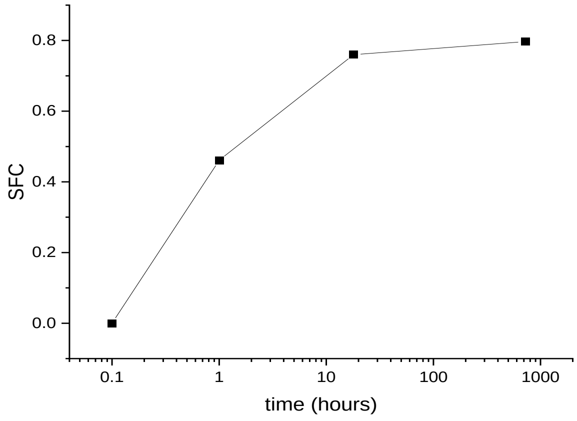

***Figure S2**. Temporal development of the SFC in BuSP.*

(1) Arita-Merino, N.; Van Valenberg, H.; Gilbert, E. P.; Scholten, E., Quantitative Phase Analysis of Complex Fats during Crystallization. *Crystal Growth and Design* **2020,** 20, (8), 5193-5202.
